# Acute intestinal pseudo-obstruction secondary to Sjogren’s syndrome in pregnancy: a case report and literature review

**DOI:** 10.1186/s12884-023-05757-5

**Published:** 2023-06-26

**Authors:** Chang Liu, Ka U Lio, Yiping Le, Chuan Wang, Xin Kang, Jianhua Lin, Yu Zhang, Ning Zhang

**Affiliations:** 1 Department of Obstetrics and Gynecology, Renji Hospital, School of Medicine, Shanghai Jiao Tong University, 160 Pujian Road, Pudong New District, 200127 Shanghai, China; 2 Department of Medicine, Temple University Hospital, Lewis Katz School of Medicine at Temple University, 3401 N Broad St, Philadelphia, PA 19140 USA

**Keywords:** Intestinal pseudo-obstruction (IPO), Sjögren’s syndrome (SjS), Pregnancy

## Abstract

**Background:**

Intestinal pseudo-obstruction (IPO) is a rare disease, and its clinical manifestations can resemble mechanical intestinal obstruction leading to unnecessary and potentially harmful surgery. Certain autoimmune diseases have been associated with IPO, however, cases secondary to Sjögren’s syndrome (SjS) are especially rare.

**Case presentation:**

We described the first case of SjS-associated acute IPO in pregnancy, which was successfully treated with combined immunosuppressive therapy and resulted in an uneventful caesarean delivery.

**Conclusions:**

Women with SjS is likely to experience more complications during pregnancy, and IPO rather than the classic symptoms could be the first sign of SjS flares. IPO should be suspected in patients with unrelenting symptoms of small bowel obstruction, and a multidisciplinary approach can provide optimal management of such high-risk pregnancies.

## Background

Intestinal pseudo-obstruction (IPO) is a rare disease characterized by gastrointestinal propulsive motility impairment and has similar clinical features to mechanical intestinal obstruction. Misdiagnosing IPO as mechanical obstruction could lead to unnecessary and potentially harmful surgery [1]. IPO tends to be secondary to other diseases, such as endocrine disorder, circulatory diseases, cancers, trauma, and autoimmune diseases including systemic lupus erythematosus (SLE), systemic sclerosis, undifferentiated connective tissue disease, and rheumatoid arthritis. However, cases secondary to Sjögren’s syndrome (SjS) are rare, and only sporadic cases have been reported [2].

Sjogren’s syndrome (SjS) is a chronic autoimmune inflammatory disorder characterized by impaired lacrimal and salivary function with resultant dryness of the eyes and mouth. Primary SjS mainly affects women in the fourth decade of life, with a prevalence rate of 3.9–5.3 per 100,000 [3]. Thus, pregnancy outcomes of women with primary Sjogren’s syndrome are limited and there is a scarcity of data on the management of SjS in pregnancy. Here, we described the first case of SjS-associated acute IPO in pregnancy, which was successfully treated with combined immunosuppressive therapy and resulted in an uneventful caesarean delivery.

## Case presentation

A 36-year-old gravida 3 para 0 at 29 weeks’ gestation presented with acute abdominal pain, nausea, and vomiting for one day without any inciting factors. She denied any associated symptoms such as fever, chills, diarrhea, or constipation. She denied consumption of raw or undercooked food or recent travel history. Her past medical history was only significant for primary SjS diagnosed four years ago, she presented with the xerostomia and dry eyes for months and then test for antinuclear antibody (ANA) and anti-Ro52 and Ro60 antibody were positive, positive Schirmer’s test and unstimulated salivary flow rate, and salivary gland biopsy showed focal lymphocytic sialadenitis with a focus score of ≥ 1 per 4 mm^2^, she was treated with daily hydroxyquinoline 200 mg since then and the disease was maintained stable until this admission. She has had two uncomplicated spontaneous miscarriage, so in the early stages of this pregnancy, prednisone 10 mg daily was use for the recurrent spontaneous miscarriage. She denied a history of surgery in the past.

On presentation, vital signs were as follow: temperature 98.2 F, blood pressure 115/67mmHg, heart rate 80 beats/min, respiratory rate 18 breaths/min, oxygen saturation 98% on room air. On exam, the abdomen was soft, non-tender, and without rigidity or rebound tenderness. Laboratory studies showed leukocytosis and neutrophilic predominance with white blood cells (WBC) count and the percentage of neutrophils fluctuating between 9.14 ~ 12.84 × 10^9^/L and 80.2 ~ 86.9%, respectively, low potassium of 2.8mmol/L, elevated erythrocyte sedimentation rate (ESR) at 40 mm/h (normal range (NR) 0–20) and elevated fibrinogen of 6.05 g/L (NR 2–4), hemoglobin was 105 g/L and the platelet count was 260 × 10^9^/L, serum albumin was 33 g/L. Other laboratory profile including liver and kidney function, thyroid function, serum electrolytes, and urinalysis were basically normal. C-reactive protein (CRP) was 0.61 mg/L (normal range (NR) 0–8) and serum complements, procalcitonin and serum ferritin were normal. Autoimmune studies were notable for an elevated antinuclear antibody (ANA) with a titer at 1:160, positive anti-Ro52 antibody of 49 (normal range < 25), and anti-Ro60 of 71 (normal range < 25). Other immunological antibodies were all negative including anti-(double stranded)-DNA antibodies, anti-β2- glycoprotein I antibody (IgG and IgM), anti-Sm antibody, anti-nRNP antibody, anti-SSB/La antibody, anti-Jo-1 antibody, anti-scl-70 antibody, anti-ribosomal P-protein antibody, anti-histone antibody, anti-nucleosome antibody, anticentromere B antibody, anti-PM-Scl antibody, anti-proliferating cell nuclear antigen antibody, and anti-mitochondria-M2 antibody. Standardized lupus ratio was 1.18 TR (NR 0.89–1.2). Immunoglobulin was detected with IgG of 11.9 g/L (NR 8.6–17.4), IgM of 1.17 g/L (NR 0.5–2.8) and IgA of 3.94 (NR 1.0-4.2) Stool examination, fecal occult blood test as well as urinalysis were normal. Complete abdominal ultrasound demonstrated no intraabdominal process or peritoneal effusion.

**Fig. 1:**
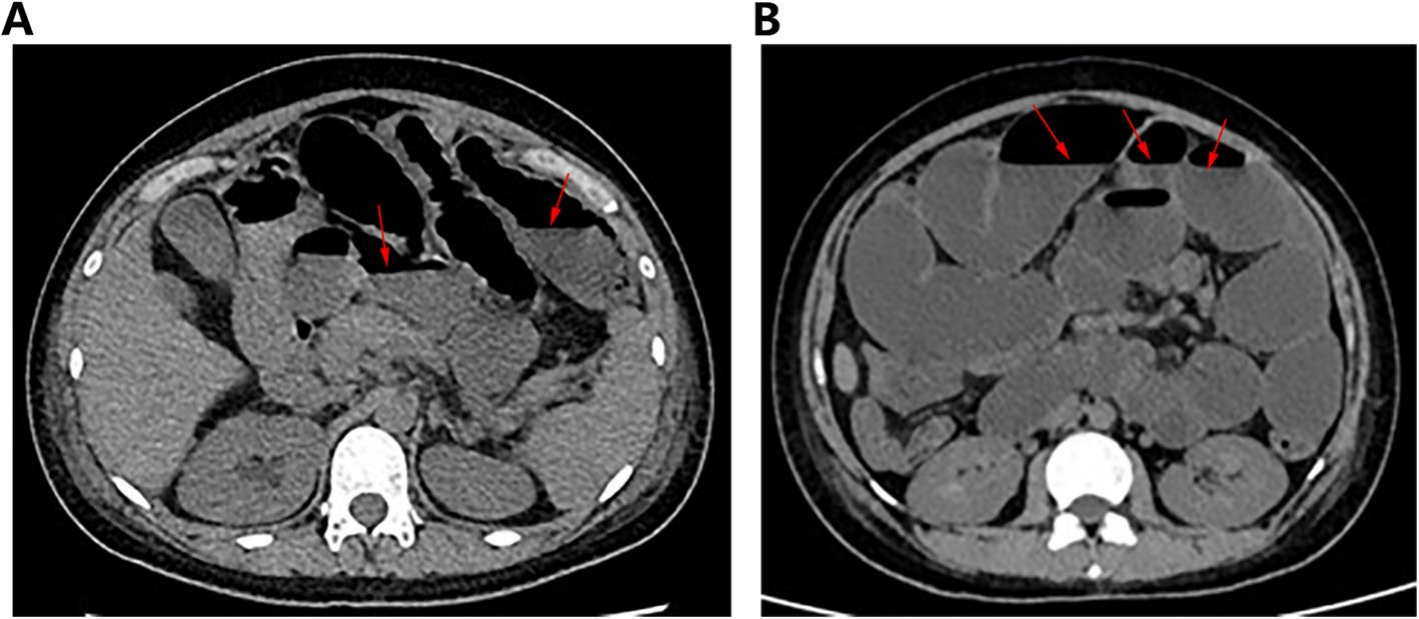
CT images show typical manifestations of intestinal obstruction. **A** the CT image for the first time revealed dilated small bowel with the widest diameter of 45 mm suggestive of small bowel obstruction; **B** the repeated CT image revealed stable dilated small bowels with the widest diameter of 53 mm, with no signs of mechanical obstruction, bowel necrosis, or perforation. Red arrows point to the typical air-fluid level of the bowel

The patient was admitted for inpatient management for an initial diagnosis of acute gastroenteritis. She received supportive measures including intravenous fluid and electrolyte repletion, and symptomatic control with bowel rest and metoclopramide. After four days of treatment, the patient developed worsening nausea, vomiting, cramping abdominal pain, and inability to pass flatus and stool, which led to the concern for intestinal obstruction. A Computed tomography (CT) of the abdomen was subsequently performed which revealed dilated small bowel with the widest diameter of 45 mm suggestive of small bowel obstruction, however, no anatomical lesions leading to obstruction were identified on CT imaging (Fig. 1A). She was treated with conservative management including nothing by mouth, intravenous fluids, and parenteral nutrition. A nasogastric tube was inserted for decompression of the gastrointestinal tract. Despite the above measures, her symptoms persisted and due to progressive deterioration, a multidisciplinary discussion including obstetricians, rheumatologists, and surgeons was held. Considering medical history, clinical presentations, laboratory, and imaging findings, we suspected acute intestinal pseudo-obstruction secondary to underlying SjS. The patient was treated with immunosuppressive therapy including intravenous methylprednisolone 80 mg daily and intravenous immunoglobin (IVIG) 20 g daily. On day two of immunosuppressive therapy, the patient began to show signs of improvement and was able to pass flatus and stool. However, she continued to experience progressive abdominal pain along with nausea and vomiting. Given concern for bowel perforation or volvulus, another CT of the abdomen was performed which revealed stable dilated small bowels with the widest diameter of 53 mm, with no signs of mechanical obstruction, bowel necrosis, or perforation (Fig. 1B).

After a multidisciplinary discussion, immunosuppressive therapy was deemed effective, and we believed the aggravated symptoms were likely attributable to increased bowel movement. We decided to continue immunosuppressive therapy and the patient completed a 5-day course of IVIG and a 7-day course of pulse dose steroids, followed by an oral prednisone taper. After treatment, her symptoms subsided and was able to advance diet.

**Fig. 2:**
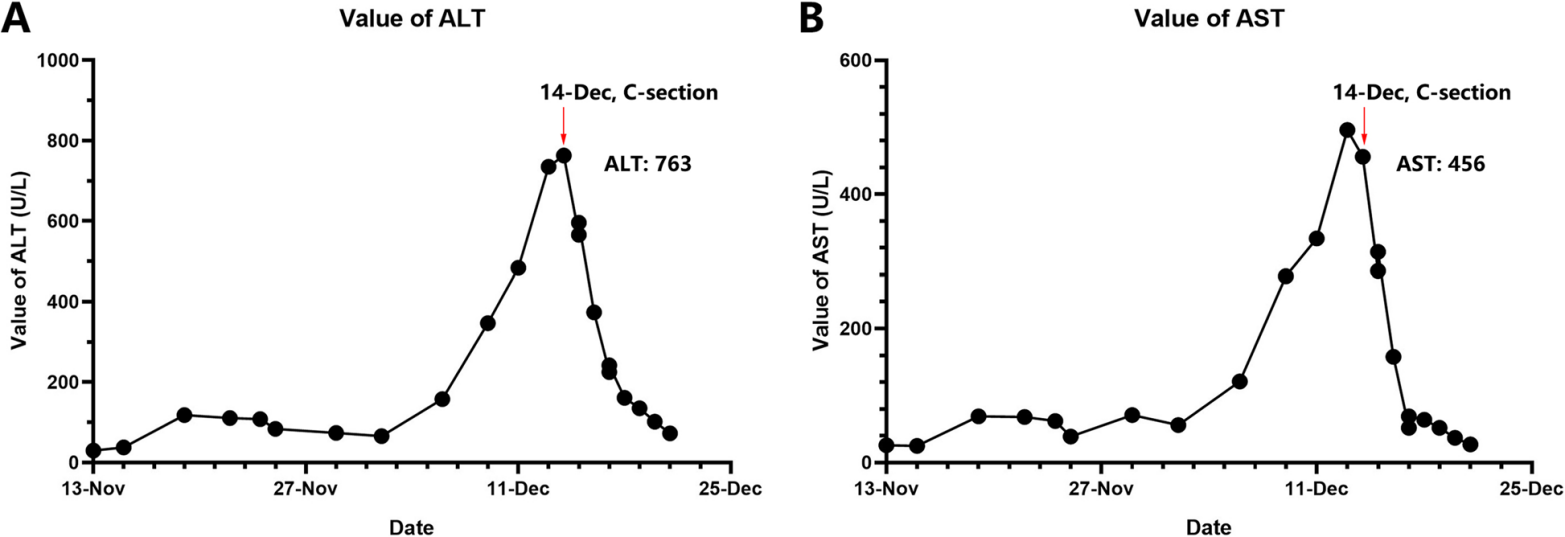
The alteration of relevant indicators of liver function in the pregnancy and post-partum. The value of ALT (A) increased from 118 to 763 U/L, AST increased from 69 to 496 U/L consistent with a hepatocellular injury pattern during pregnancy, and gradually downtrended to the normal range after cesarean section. ALT, Alanine aminotransferase; AST, Aspartate aminotransferase; C-section, Cesarean section

**Fig. 3:**
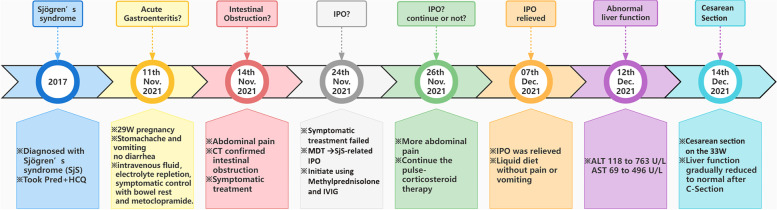
Schematic diagram of the progression of the disease of Sjögren’s syndrome (SjS). Pred, Prednisone; HCQ, hydroxychloroquine; IPO, Intestinal pseudo-obstruction; MDT, Multi-DisciplinaryTreatment; ALT, Alanine aminotransferase; AST, Aspartate aminotransferase; IVIG, Intravenous Immunoglobulin

Unfortunately, the patient developed signs of acute liver injury with rising aminotransferases after the resolution of IPO. ALT increased from 118 to 763 U/L, and AST increased from 69 to 496 U/L consistent with a hepatocellular injury pattern (Fig. 2). Other biomarkers, including bilirubin, total bile acid, albumin, and coagulation studies were within a relatively normal range. Blood pressure, hemoglobin and platelet remained stable and there were no signs of hemolysis. The acute hepatitis panel and autoimmune liver disease antibody spectrum were negative, including antimitochondrial antibody (AMA). A meticulous evaluation was negative for other etiologies of acute liver injury such as autoimmune hepatitis, viral hepatitis, HELLP syndrome (Hemolysis, Elevated Liver enzymes and Low platelets Syndrome), AFLP (Acute Fatty Liver of Pregnancy), and ICP (Intrahepatic Cholestasis of Pregnancy), and we attributed it to Sjogren’s syndrome or progressed gestation. After consideration of both risks and benefits, the patient proceeded with an urgent caesarean section at 33 weeks’ gestation which resulted in an uneventful delivery of a neonate weighed 1850 g. Apgar’s score was both 10 at 1 and 5 min. On post-partum day 4, AST recovered to the normal range and ALT rapidly downtrended to 225 U/L and then recovered to normal on post-partum day 7 (Fig. 2), and the patient was eventually discharged home on post-partum day 7. The schematic diagram of the progression of the patient’s disease was showed in Fig. 3. On 6-month and 12-month follow-up, her was regularly checked by Rheumatologist, her overall condition was satisfactory without any systemic manifestations and hadn’t suffered from any gastrointestinal symptoms since the pregnancy termination.

## Discussion and conclusions

Women with SjS are likely to experience more complications during pregnancy [4]. Intestinal pseudo-obstruction (IPO) is a disorder characterized by colonic dilatation in absence of an anatomic lesion that obstructs the flow of intestinal contents, and most patients present with signs and symptoms of mechanical obstruction including abdominal pain, nausea, and vomiting. IPO may be idiopathic or secondary to a variety of diseases, including neurologic disorders (e.g., Parkinson disease, spinal cord injury), metabolic/endocrine disorders (e.g. diabetes mellitus), paraneoplastic disorders, and autoimmune diseases [1]. H. Ohkubo et al. concluded a total of 160 cases of IPO and 41 cases (25.6%) were secondary to another disease, including systemic sclerosis (23 cases, 56.1%), dermatomyositis (4 cases, 9.8%), mixed connective tissue disease (MCTD) (3 cases, 7.3%) and SjS (1 case, 2.4%) [5]. IPO secondary to SjS is a clinical rarity, and evidence was limited to case reports [2]. We conducted a comprehensive literature review by searching the Medline, Embase, and Google Scholar databases to identify all English literature published using the keywords “intestinal pseudo-obstruction or IPO”, “pregnancy”, “Sjogren’s syndrome or SS, SjS”, “gestation”, alone or in combination. We reviewed all articles (including case reports, case series, and review articles), and demonstrated that the current patient is the first case of SjS-associated acute IPO in pregnancy, which was successfully treated with combined immunosuppressive therapy and resulted in an uneventful caesarean delivery.

Our patient initially presented with non-specific symptoms of abdominal pain, nausea, and vomiting without any inciting factors, and she was treated for acute gastroenteritis. However, her symptoms continued to worsen despite supportive measures which eventually led to the diagnosis of intestinal obstruction. Diagnosis of IPO is mainly established by abdominal CT scan, however, in most cases remains difficult to definitively distinguish between IPO and mechanical obstruction [5]. In our case, we decided to proceed with conservative management as there were no signs of imminent perforation, ischemia, or necrosis. In addition, clinicians should be mindful of identifying any secondary causes of IPO, as this could change the management approach as shown in the present case. We believed IPO was driven by the patient’s underlying autoimmune diseases and the initiation of immunosuppressive therapy eventually led to the resolution of IPO.

What made it a diagnostic challenge was that the clinical picture in the present case was not suggestive of SjS disease flares, as patient did not manifest with any specific signs or symptoms of SjS and the results of autoimmune studies were unremarkable. In fact, there were case reports of IPO as the first manifestation of Sjogren’s syndrome [2], including other autoimmune diseases such as SLE [6–9], connective tissue disease [1], and primary intestinal autoimmune disease [10].

The goal of management IPO is to decompress bowels in order to minimize the risk of colon perforation and ischemia as well as effective control of underlying cause. There is a scarcity of data on the management of primary SjS in pregnancy. According to the British Society for Rheumatology (BSR), primary SjS with systemic complications, recommended immunomodulatory therapy included: (1) corticosteroids; (2) DMARDs (Disease-Modifying Antirheumatic Drug) such as hydroxychloroquine, azathioprine, methotrexate, Mycophenolate etc.; (3) Biologics: Rituximab; (4) Other potential treatments: IVIG, colchicine, dapsone, topical tacrolimus [11]. However, some immunosuppressive medications may cross the placenta and cause fetal harm. Thus, the benefits of treatment during pregnancy must be weighed against the risk of Sjogren activity having a delirious effect on the mother and the fetus. The rare case report of IPO related to SjS indicated that glucocorticoid and immunosuppressive agents could be administrated as the principle treatment, accompanied by symptomatic treatments to prevent the progression to intestinal necrosis and perforation [2]. Similarly, for SLE-related GI manifestations, it’s recommended that immunosuppressive therapy should be tailored to the severity of organ involvement, for potentially organ-threatening or life-threatening GI involvement, high-dose corticosteroids ranging from 40 mg/d to 1 mg/kg/d are indicated [12]. Thus, based on the general guideline for autoimmune disease with systemic features as well as the experiences of published successful case reports, in the first case of SjS-associated acute IPO in pregnancy, the symptoms were successfully treated by combined immunosuppressive therapy of pulse dose steroids, IVIG and hydroxychloroquine which are relatively safe for maternal and fetal safety.

Although the cause of the liver damage has not been clearly identified, both SjS and pregnancy could be the cause of acute liver injury. SjS is associated with various hepatic abnormalities, including transaminitis, primary biliary cholangitis, or autoimmune hepatitis. The patient excluded the possibility of hepatotoxic drugs, fatty liver disease, fatty liver disease, and chronic viral infections, but there is still a possibility of Sjogren’s syndrome inducing autoimmune liver disease [13]. SjS induced primary biliary cirrhosis (PBC) or sclerosing cholangitis were also considered but eventually excluded after a meticulous evaluation. Hepatic fibrosis may be induced by SjS and lead to abnormal liver function [14], but due to the unavailability of liver biopsy during pregnancy to confirm the occurrence of fibrosis, SjS induced liver fibrosis is not the primary cause of consideration in this case. Given the fact that liver enzymes normalized after pregnancy termination, pregnancy itself was more likely to be the cause of hepatic injury, rather than Sjögren’s syndrome. As shown in the previous research, women with SjS were more likely to have premature birth [3, 15, 16]. Our patient presented the initial symptoms of SjS-associated IPO at gestation of 29 weeks, and was successfully treated with combined immunosuppressive therapy by multidiscipline team resulting in an uneventful caesarean delivery at 33 weeks’ gestation.

This is the first case of SjS-associated acute IPO in pregnancy. IPO should be suspected in patients with unrelenting symptoms of small bowel obstruction and it could present as the first sign of disease flares of underlying diseases. Clinicians should meticulously explore secondary causes of IPO, and this could affect the management approach. Women with SjS are likely to experience more complications during pregnancy and a multidisciplinary approach involving obstetricians, rheumatologists and pediatricians can provide optimal management of such high-risk pregnancies.

## Data Availability

The relevant materials during the current study are available from the corresponding author (Ning Zhang, email: ningning1723@126.com) upon reasonable request.

## References

[1] Di Nardo G, Di Lorenzo C, Lauro A, Stanghellini V, Thapar N, Karunaratne TB, Volta U, De Giorgio R (2017). Chronic intestinal pseudo-obstruction in children and adults: diagnosis and therapeutic options. Neurogastroenterol Motil.

[2] Jia JT, Wei H, Li H (2012). Primary Sjögren’s syndrome accompanied by intestinal obstruction: a case report and literature review. Chin Med Sci J.

[3] Mariette X, Criswell LA (2018). Primary Sjögren’s syndrome. N Engl J Med.

[4] Upala S, Yong WC, Sanguankeo A (2016). Association between primary Sjögren’s syndrome and pregnancy complications: a systematic review and meta-analysis. Clin Rheumatol.

[5] Ohkubo H, Iida H, Takahashi H, Yamada E, Sakai E, Higurashi T, Sekino Y, Endo H, Sakamoto Y, Inamori M (2012). An epidemiologic survey of chronic intestinal pseudo-obstruction and evaluation of the newly proposed diagnostic criteria. Digestion.

[6] Wang JL, Liu G, Liu T, Wei JP (2014). Intestinal pseudo-obstruction in systemic lupus erythematosus: a case report and review of the literature. Medicine.

[7] Sultan SM, Ioannou Y, Isenberg DA (1999). A review of gastrointestinal manifestations of systemic lupus erythematosus. Rheumatology (Oxford).

[8] Perlemuter G, Chaussade S, Wechsler B, Cacoub P, Dapoigny M, Kahan A, Godeau P, Couturier D (1998). Chronic intestinal pseudo-obstruction in systemic lupus erythematosus. Gut.

[9] Kim HJ, Park JY, Kim SM, Woo YN, Koh BH, Cho OK, Ko YH, Park MH (1995). Systemic lupus erythematosus with obstructive uropathy. Case report and review. J Korean Med Sci.

[10] Ghirardo S, Sauter B, Levy G, Fiel IM, Schiano T, Gondolesi G (2005). Primary intestinal autoimmune disease as a cause of chronic intestinal pseudo-obstruction. Gut.

[11] Price EJ, Rauz S, Tappuni AR, Sutcliffe N, Hackett KL, Barone F, Granata G, Ng WF, Fisher BA, Bombardieri M (2017). The British Society for Rheumatology guideline for the management of adults with primary Sjögren’s syndrome. Rheumatology (Oxford).

[12] Brewer BN, Kamen DL (2018). Gastrointestinal and hepatic disease in systemic lupus erythematosus. Rheum Dis Clin North Am.

[13] Zeron PB, Retamozo S, Bové A, Kostov BA, Sisó A, Ramos-Casals M (2013). Diagnosis of liver involvement in primary Sjögren syndrome. J Clin Transl Hepatol.

[14] Androutsakos T, Voulgaris TA, Bakasis AD, Koutsompina ML, Chatzis L, Argyropoulou OD, Pezoulas V, Fotiadis DI, Papatheodoridis G, Tzioufas AG (2022). Liver fibrosis in primary Sjögren’s syndrome. Front Immunol.

[15] Brito-Zerón P, Baldini C, Bootsma H, Bowman SJ, Jonsson R, Mariette X, Sivils K, Theander E, Tzioufas A, Ramos-Casals M (2016). Sjögren syndrome. Nat Rev Dis Primers.

[16] Fox RI (2005). Sjögren’s syndrome. Lancet.

